# Development and validation of a scale for the assessment of the knowledge-attitude-practice of parents towards children snoring

**DOI:** 10.1186/s12875-024-02360-1

**Published:** 2024-04-08

**Authors:** Siyan Guo, Xiaoyue Hu, Xiaokai Wang, Hongyan Tie, Qiujun Zhang, Caixia Li, Luying Qin, Hongxia Su

**Affiliations:** 1 School of Nursing and Health, Zhengzhou University, No. 100, Science Avenue, Zhengzhou, Henan China; 2 School of Basic Medical Sciences, Zhengzhou University, Zhengzhou, Henan China; 3 No.5 Affiliated Hospital of Zhengzhou University, Zhengzhou, Henan China; 4 Zhengzhou Central Hospital, Zhengzhou, Henan China; 5 No.1 Affiliated Hospital of Zhengzhou University, Zhengzhou, Henan China

**Keywords:** Snoring, Child, Instrument

## Abstract

**Background:**

Children Snoring is a common childhood disorder that affects the growth and development of children and is detrimental to their health. Increasing awareness of Children Snoring among parents is important.

**Aim:**

To develop the Knowledge-Attitude-Practice of Parents towards Children Snoring Scale and test the reliability and validity of the scale.

**Methods:**

The development of the tool was divided into two phases involving 1257 parents from China. In the first phase, an initial project bank was created through a literature review. This was followed by a Delphi expert consultation, group discussion and pre-survey. The second stage screened the items and conducted an exploratory factor analysis, then conducted a confirmatory factor analysis and tested for reliability and validity.

**Results:**

Support was found for the 25-item Knowledge-Attitude-Practice toward Children Snoring scale. Exploratory and confirmatory factor analyses provide support for four subscales: (parental basic cognition toward Children Snoring; parents’ perception of complications of Children Snoring; parents’ attitude towards Children Snoring; parents’ concern and prevention of Children Snoring). Internal consistency for the total scale was high (Cronbach’s α = 0.93). The intraclass correlation coefficient of test-retest reliability was 0.92 (95%CI: 0.85 to 0.95), which provided support for the stability of the scale.

**Conclusion:**

The Knowledge-Attitude-Practice of Parents towards Children Snoring scale shows promise as a measure that may be used by medical workers and community children’s health managers.

## Background

Snoring, a mild sleep breathing disorder, is a common nighttime disorder in school-aged children, with 27% of children suffering from snoring [1]. Children Snoring is triggered by turbulent airflow and vibrations of the soft tissues, and the main causative factors include adenoid hypertrophy, enlarged tonsils, acute and chronic inflammation of the nasal cavity, obesity, as well as developmental craniofacial anomalies [2–5]. Without timely treatment and behavioral intervention, snoring will develop into obstructive sleep apnea-hypopnea syndrome (OSAHS) that threatens children’s health seriously in the future [6, 7]. OSAHS can lead to a series of secondary complications in children, such as left heart failure, growth retardation, endocrine disorders, otitis media, chronic respiratory diseases, the risk of cognitive deficits, etc [8–14].

Parents are typically the first individual to recognize the signs of Children Snoring. Studies have shown that parents lack basic knowledge of Children Snoring, which can lead to delays in seeking medical attention [15–17]. Studies shows that more than one-third of children who snore develop obstructive sleep apnoea at four-year follow-up, while early intervention for snoring in children can significantly improve snoring and prevent OSAHS [7, 18]. However, 80% of parents are unaware of the cardiopulmonary diseases and developmental delays that can result from pediatric snoring [19]. A study shows that over 80% of parents in the community are eager to learn about the symptoms and consequences for children’s snoring [20]. However, there is no scientific tool about parents’ knowledge, attitude, and practice regarding Children Snoring. Existing assessment tools for children’s snoring are aimed at diagnostic screening purposes [21–24], and the few questionnaires on knowledge of children snoring are designed for use by primary care physicians,, pediatricians, or dentists [25–27]. These assessment tools have the advantage of quickly screening for children at high risk of snoring or OSA and investigating the extent to which doctors are well informed about children’s sleep disorders. However, the limitation is that they ignore the need of children’s parents about childhood snoring. The Knowledge-Attitude-Practice (KAP) theory, also known as the Knowledge, Attitude, Beliefs, and Behaviour model (KABP), posits that knowledge and information are the basis for the generation of health beliefs and attitudes and that health behaviors are further generated based on health beliefs and attitudes [28]. The theory explains the dialectical relationship between knowledge, attitude and practice, which helps the researcher to investigate the current status of children’s parents’ knowledge, attitude and then practice of children’s snoring, and to further explore the knowledge, attitude and practice gaps that exist among children’s parents in relation to children’s snoring. Based on the Knowledge-Attitude-Practice (KAP) theory, we have developed the Knowledge-Attitude-Practice Scale of Parents towards Children Snoring, which will provide important health guidance for children’s parents, and also provide a novel scientific evaluation tool for medical workers and community children’s health managers.

## Methods

### Phase 1 developing items for the scale

#### Literature research

**Fig. 1:**
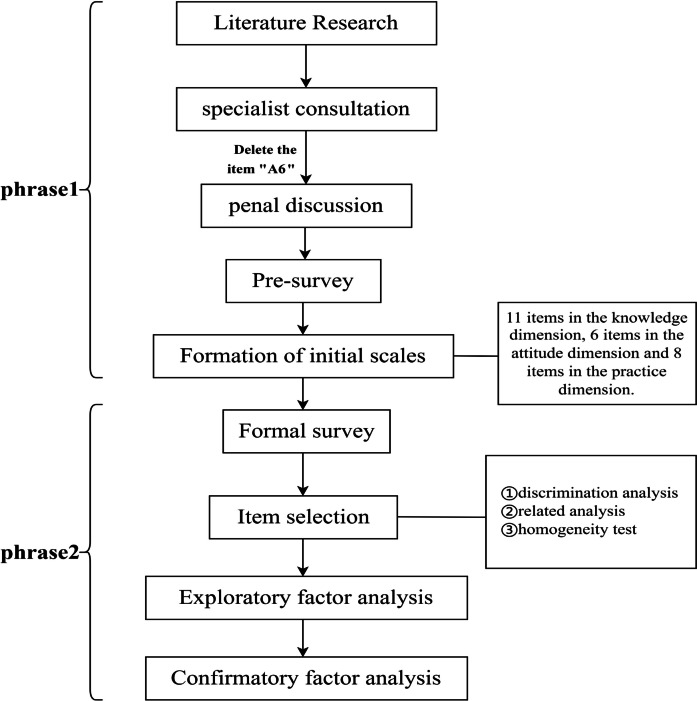
Flowchart of the study

During the scale development phase, the scales were developed with “child”, “children”, “pediatric”, “snore”, “snoring”, “OSA”, “OSAHS”, “KAP”, “knowledge”, “attitude”, “practice”, “scale”, “questionnaire”, “assessment”, “tool”, “measure” were used as search terms to search literature in databases including CNKI, PubMed, Scopus, Embase and Web of Science. The search date for the literature is before May 2022. Excluding duplicate publications and incomplete content, 56 articles were eventually included. The steps of this research are shown in Fig. 1.

#### Specialist consultation

We assembled a panel of four ear-nose-throat (ENT) chief physicians from the Children’s Hospital, two ENT nurse practitioners, three ENT deputy nurse practitioners two chief physicians from the Respiratory Sleep Centre, and a nurse in the respiratory sleep unit. The experts were selected based on the following criteria: (1) master’s degree or higher; (2) more than ten years of work in the field; (3) informed voluntary participation in this study. All of the above specialists were from tertiary hospitals. The Delphi correspondence method was used to obtain expert opinions on the scale through consultation [29].

The expert letter inquiry questionnaire consisted of three parts. The first part was a guide to the inquiry questionnaire, which included the requirements for completing the questionnaire, the important elements of the questionnaire, how and when to return the questionnaire, and acknowledgments. The second part contained general information about the expert. It includes the age, gender, education, title, administrative position, nature of work, years of work, work unit, contact telephone number, and email address of the expert. This part of the information was mainly collected to analyze the degree of authority of the experts consulted.

The third and most important part of the questionnaire is the revision of the content and language descriptions of the items in the pool. The importance of the indicators in the questionnaire was judged on a 5-point scale, with ratings of “very important”, “important”, “average”, “unimportant” and “very unimportant” (with scores of 5, 4, 3, 2, and 1 respectively). The paper questionnaire was collected within 14 days after the questionnaire was sent out and the results were then statistically analyzed by the research team members. Experts were asked to judge whether: (a) the items were in line with the content of the Children Snoring; (b) the items were reflected the content of the corresponding dimensions; (c) the content of the assessment was repetitive; (d) the language was concise and clear; (e) there was any ambiguity; (f) the items needed to be combined. If the expert has new items that are suitable for the assessment of children’ s snoring, he/she may also suggest adding the corresponding items. Content validity refers to the appropriateness of the scale for the content to be measured, i.e., the appropriateness and consistency of the content to be measured, and is calculated using expert responses to each item, and is represented by the scale-level content validity index (S-CVI) and the average item-level content validity index (I-CVI), which is calculated as the mean of the S-CVI [30]. “A6” was deleted.

#### Panel discussion

The wording of the scale items was discussed by a discussion group consisting of three chief otolaryngologists from tertiary hospitals, three nurse practitioners from tertiary hospitals, and two current Master of Nursing students. This phase aimed to revise the wording of the items to make them easy to understand.

The discussion group discussed the scales in three rounds of one hour each until all group members reached an agreement. The Discussion Group made changes and additions to the wording of “A1”, and “P7”. Replace “A1: Parents should pay close attention to their children when they sleep” with “A1: Parents should pay close attention to whether their children snore while sleeping”. Replace “P7: Care for your child’s diet” with “P7: Arrange child’s diet and nutrition scientifically.”.

#### Pre-survey

A convenience sample of 40 parents of children was selected from an area of Zhengzhou City, Henan Province, China, based on the following inclusion criteria: (a) having at least one child under 14 years old; (b) having primary school education and above; (c) voluntary participation and cooperation in this survey. Exclusion criteria: (a) those who hadn’t lived with their children for more than 6 months; (b) those who had mental or physical illness that prevented them from completing the questionnaire correctly. The final 38 parents of children participated in the pre-survey and all scales were completed anonymously. Once the scales were completed, parents were asked about the appropriateness of the scale and how they felt when completing every item of the scale. Parental feedback on “child behavior problems” in K9 and “hearing loss” in P3 required examples for the subjects to understand. Subsequently, adjustments were made based on parent feedback. To calculate the Test-retest reliability of the scale, the scale was administered again to 38 parents 2 weeks after the initial survey.

### Phase 2 evaluation of the scale’s psychometric properties

#### Tools

Three dimensions were present in the initial scale: parents’ knowledge, attitude, and practice toward Children Snoring. The knowledge dimension includes 11 items on the classification, causes, and adverse effects of Children Snoring; the attitude dimension includes 6 items about the parents’ attitude of noticing children’s breathing status, timely diagnosis and treatment, and scientific diet and weight control; and the practice dimension consists of 8 items on the seeking medical attention when children have relevant symptoms, observing children’s snoring symptoms, paying attention to children’s nutrition, and monitoring children’s weight control. The answers were given on a 5-point Likert-type scale, with 1 for “Don’t know at all”, 2 for “Don’t know”, 3 for “Not sure”, 4 for “Partially know” and 5 for “Know all” in the “Knowledge” dimension, and the options in the “Attitude” and the “Practice” dimension being “Strongly disagree” “Disagree” “Not sure” “Agree”, “Strongly agree” respectively.

#### Participants

We used a stratified sampling method to draw 1270 parents of children under 14 from all districts in Henan Province, China between 1 September 2022 and 30 November 2022. Random numbers were then generated in Stata software to split the total samples into two random subgroups. One was then used to screen items and conduct exploratory factor analysis (EFA) while the other was used for confirmatory factor analysis (CFA). Inclusion and exclusion criteria were the same as pre-survey.

#### Statistical methods

Items were first screened by the critical ratio value method, correlation analysis, and homogeneity tests. The scale dimensions were then explored through exploratory factor analysis. Confirmatory factor analyses were used to check the fit of the model to the data. The scale was evaluated through internal consistency reliability, construct validity, content validity, convergent validity and discriminant validity.

## Results

### Characteristics of the study participants

A total of 1270 questionnaires were distributed and 1257 effective questionnaires were eventually returned, representing a 98.9% return rate.

The participants were composed of 71.6% aged between 31 and 40 years old, 50.6% of the participants had heard of children snoring, 56.8% of the participants had 2 children at home, 29.7% of the participants had one child at home, 65.0% of participants live in the city. The educational level of the participants was mostly high school/technical school.

This sample was randomly divided into two samples, one for screening items and exploratory factor analysis (*n* = 629); and the other for confirmatory factor analysis, reliability, and validity (*n* = 628). Chi-square tests showed no statistically significant differences between the two subgroups with respect to demographic variables (Table 1)including: identity (*X* ^2^ = 2.000, *P* = 0.157), residence (*X* ^2^ = 1.639, *P* = 0.200), age (*X* ^2^ = 2.863, *P* = 0.413), education level (*X* ^2^ = 2.692, *P* = 0.611), number of children in the family (*X* ^2^ = 0.361, *P* = 0.835), ever heard of children snoring (*X* ^2^ = 0.001, *P* = 0.978) and is there a snoring child in the family (*X* ^2^ = 0.149, *P* = 0.699).

**Table 1:** Socio-demographic characteristics of participants

General information	EFA Subsample n(%)	CFA Subsample n(%)	***X*** ^**2**^	***P***
Identity				
Father	136(21.6)	111(17.7)	2.000	0.157
Mother	493(78.4)	517(82.3)		
Age				
≤20	12(1.9)	11(1.8)	2.863	0.413
21-30	61(9.7)	61(9.7)		
31-40	461(73.3)	439(69.9)		
≧41	95(15.1)	117(18.6)		
Residence				
Urban	398(63.3)	419(66.7)	1.639	0.200
Countryside	231(36.7)	209(33.3)		
Education level				
Primary school	5(0.8)	7(1.1)	2.692	0.611
Junior high school	110(17.5)	119(18.9)		
Senior high school/Technical school	200(31.8)	205(32.7)		
Junior college	199(31.6)	174(27.7)		
Bachelor's degree/ above	115(18.3)	123(19.6)		
Number of children in the family				
1	182(28.9)	191(30.4)	0.361	0.835
2	360(57.2)	354(56.4)		
≧3	87(13.8)	83(13.2)		
Ever heard of Children Snoring				
Yes	319(50.7)	318(50.6)	0.001	0.978
No	310(49.3)	310(49.4)		
Is there a snoring child in the family?				
Yes	148(23.5)	142(22.6)	0.149	0.699
sNo	481(76.5)	486(77.4)		

*EFA *Exploratory Factor Analysis, *CFA *Confirmatory Factor Analysis

### Item selection

#### (a) discrimination analysis

All subjects were ranked from highest to lowest according to total scores on the scale, with those in the top 27% of scores included in the high-score group and those in the bottom 27% included in the low-score group. An independent sample t-test revealed significant differences between the high-score group and low-score group for each item, which indicated that all items in the scale had good discriminatory power and that there was no need to exclude any item.

#### (b) related analysis

In this study, the correlation coefficients between each item’s score and the total score of the scale all exceed 0.30 (*p* < 0.001), indicating good discrimination between items [31], without being too high (i.e., > 0.85) which can indicate multicollinearity [32]. As shown in Table 2, in this study all correlation coefficients was between 0.30 and 0.80(*p* < 0.001), so there was no need to remove any items. The mean inter-item correlation was 0.58 for the knowledge dimension, 0.45 for the attitude dimension, and 0.52 for the practice dimension. The mean inter-item correlations between the three dimensions were 0.19 (knowledge and attitude), 0.29 (knowledge and practice), and 0.19 (attitude and practice), respectively; indicting good item reliability [33–35].

**Table 2:** Means, ITC, Cronbach’s α, factor loadings, CR and AVE for each item

Scale	Item		Mean ± SD	ITC	Item deleted α	Cronbach’s α	Explained variance (%):	Factor loading				CR	AVE
								18.4	18.1	14.7	11.9		
Sub-scale1	K1	Snoring in children during sleep is a disease	3.71 ± 1.00	0.57	0.928	0.929		0.75				0.86	0.50
	K2	Children snoring needs medical treatment.	3.52 ± 1.10	0.66	0.926			0.73					
	K3	Knowing which hospital department to visit for children snoring.	3.21 ± 1.20	0.62	0.927			0.68					
	K4	Obesity is a cause of snoring in children.	3.58 ± 1.07	0.58	0.928			0.70					
	K5	Adenoid hypertrophy and chronic tonsillitis are popular cause of children snoring.	3.43 ± 1.17	0.67	0.926			0.67					
	K6	Nasal disorders (e.g. sinusitis, deviated septum, nasal polyps, etc.) are a cause of snoring in children.	3.43 ± 1.12	0.66	0.927			0.72					
Sub-scale2	K7	Children snoring can affect intelligent development.	3.16 ± 1.18	0.71	0.926	0.927			0.79			0.91	0.67
	K8	Children snoring can affect the development of facial features.	3.16 ± 1.22	0.7	0.926				0.80				
	K9	Children snoring can lead to behavioural problems (e.g. social withdrawal, aggression, hyperactivity, etc.).	2.91 ± 1.16	0.7	0.926				0.84				
	K10	Children snoring can lead to headaches, fatigue, poor concentration in class and memory loss.	3.17 ± 1.20	0.7	0.926				0.81				
	K11	Children snoring can lead to otitis media (fluid in the middle ear, hearing loss).	2.89 ± 1.16	0.7	0.926				0.84				
Sub-scale3	A1	Parents should pay close attention to whether their children snore while sleeping.	4.69 ± 0.73	0.34	0.931	0.931				0.61		0.87	0.52
	A2	The child’s weight needs to be kept within normal limits.	4.71 ± 0.70	0.39	0.931					0.64			
	A3	Children Snoring needs to be improved as soon as possible.	4.73 ± 0.69	0.36	0.930					0.76			
	A4	Take the initiative to seek medical advice or consultation if you notice your child snore.	4.6 ± 0.70	0.48	0.929					0.79			
	A5	If the doctor recommends specialist treatment for Children Snoring, do you agree?	4.58 ± 0.76	0.46	0.929					0.78			
	A7	Hope that hospitals, communities, schools and the media will widely disseminate the health information about Children Snoring.	4.64 ± 0.70	0.33	0.931					0.74			
Sub-scale4	P1	Pay attention to child’s breathing during sleep.	4.09 ± 0.96	0.56	0.928	0.928					0.65	0.90	0.53
	P2	If child develops nasal congestion (poor nasal ventilation), the child will be taken to a doctor.	4.10 ± 1.02	0.57	0.928						0.74		
	P3	If child snore while sleeping, the child will be taken to a doctor.	3.71 ± 1.25	0.66	0.927						0.72		
	P4	Pay attention to the child’s mental state during the day and his or her concentration while studying.	3.99 ± 1.03	0.62	0.928						0.80		
	P5	Pay attention to whether child has hearing loss (e.g. TV turned up loud).	4.03 ± 1.00	0.58	0.928						0.80		
	P6	Pay attention to child’s upper jaw and upper lip for significant outward protrusion.	3.86 ± 1.14	0.67	0.927						0.73		
	P7	Arrange child’s diet and nutrition scientifically.	4.12 ± 0.92	0.66	0.929						0.74		
	P8	Supervise and urge child to take physical exercise everyday.	4.17 ± 0.91	0.71	0.929						0.67		
**Total**		-	101.67 ± 15.85	-	-	**Overall Cronbach’s α = 0.93**	-	-	-	-	-	-	-

Sub-scale1: parental basic cognition toward Children Snoring; Sub-scale2: parents’ perception of complications of Children Snoring; Sub-scale3: parents’ attitude towards Children Snoring; Sub-scale4: parents’ concern and prevention of Children Snoring. *ITC *Item-total correlation, *CR *Composite reliability, *AVE *The average variance extracted for each dimension

#### (c) homogeneity test

As shown in Table 2, deleting any item did not result in a significant improvement in the scale’s total Cronbach’s α coefficient, indicating that the internal consistency of the scale is excellent and there is no need to delete any of the items.

### Exploratory factor analysis

The scale structure was explored through EFA consisting of principal component extraction and varimax rotation; analyses were conducted in SPSS24.0. Before conducting the factor analysis, the KMO test and Bartlett’s sphericity test were used for the suitability analysis. Factors and items meeting the following criteria will be retained: (a) Eigenvalue > 1; (b) Total variance explained > 50%; (c) Factor loadings > 0.50. Bartlett’s test of sphericity was significant (x^2^ = 10196.20, df = 325, *p* < 0.001) and the KMO value was 0.93(>0.80), indicating that the data were suitable for factor analysis [36]. Exploratory factor analysis yielded four factors that explained 63.3% of the variance and each factor contains more than two items. The results of the exploratory factor analysis based on the model of KAP theory consisting of three factors showed that the cumulative variance contribution of the three factors to the model was 51.3%. After the panel discussion and soliciting expert advice, the scale finally retained four factors, namely “parental basic cognition toward Children Snoring”, “parents’ perception of complications of Children Snoring”, “parents’ attitude towards Children Snoring” and “parents’ concern and prevention of Children Snoring”; The two sub-dimensions of “parental basic cognition toward Children Snoring” and “parents’ perception of complications of Children Snoring” together form the “parents’ knowledge of Children Snoring” dimension. The loading values for each item are shown in Table 2.

### Confirmatory factor analysis

The preliminary four-factor model based on KAP theory was tested using CFA; analyses were conducted in AMOS23.0. CFA allows investigators to specify a hypothesized factor structure in advance and then test it, thereby determining how well the proposed model fits the data. The initial model was not a good fit for the data; adjustments to the model were made based on modification indices. The results of every modification are shown in Table 3.

**Table 3:** Model fitting indexes of the knowledge-attitude-practiceof parents towards children snoring scale

Item	χ2/df	GFI	CFI	NFI	RMSEA
Initial fit	3.85	0.88	0.91	0.89	0.07
The first modification	3.46	0.89	0.93	0.91	0.06
The second modification	3.19	0.89	0.94	0.91	0.06
**The third modification**	**2.99**	**0.90**	**0.94**	**0.92**	**0.06**

χ2/df: values between 1and 3 indicate a good model fit. GFI: Adjusted goodness of fit index; values ≥ 0.90 indicate a good model fit. *CFI *Comparative fit index; values ≥ 0.90 indicate a good model fit, *NFI *Normed fit index; values ≥ 0.90 indicate a good model fit, *RMSEA *Root mean square error of approximation; values < 0.06 indicate a good model fit

**Fig. 2:**
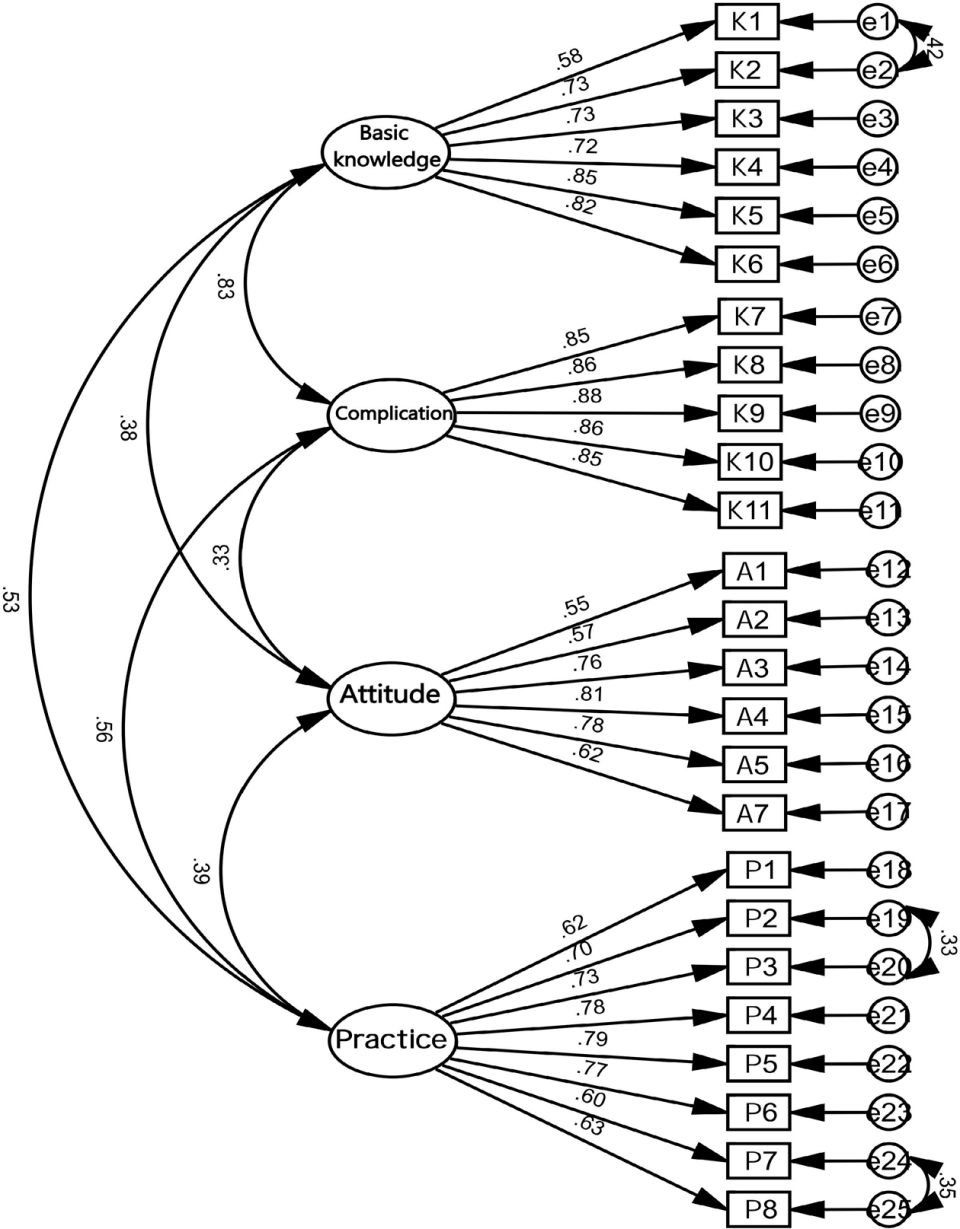
Confirmatory factor analysis of the scale

The goodness-of-fit indices were x² = 796.90; df = 266; x²/df = 2.99; RMSEA = 0.06; CFI = 0.94; NFI = 0.92; GFI = 0.90. The literature on structural variance suggests x²/df less than 3, values of RMSEA less than 0.06, and CFI、NFI、TLI greater than 0.90 the model fits well [37]. The model fit is shown in Fig. 2.

### Reliability

#### (a) Internal consistency reliability

We used Cronbach’s α to assess the consistency and stability of the instrument. A Cronbach’s α value > 0.80 usually indicates good internal consistency [38].

After retaining two decimals, the overall Cronbach’s α was 0.93 (Table 2), which indicated that the scale has good internal reliability.

#### (B) test-retest reliability

We used intra-class correlation coefficient to indicate the stability of a scale, and a scale is considered stable when the intra-class correlation coefficient for Test-retest reliability is greater than 0.75 [34, 39]. To calculate the Test-retest reliability of the scale, the scale was administered again to 38 parents 2 weeks after the initial survey.

The intra-class correlation coefficient of the scale is 0.92(95%CI:0.85 ~ 0.95), which indicates that the scale has good Test-retest reliability.

#### (C) composite reliability

Table 3 shows that all the Composite Reliability (CR) values of four factors exceed 0.80. Previous research proved that CR values > 0.80 are excellent for the evaluation of the scale combination reliability [40]. Combining Cronbach’s α and CR, the Knowledge-Attitude-Practice Scale of Parents scale has a high level of reliability.

### Validity

#### (a) content validity

The content validity of the scale is assessed through a content validity index (CVI) derived by experts. Each expert was asked to assess the relevance of each item to its corresponding dimension. The CVI of the scale (S-CVI) was calculated as the mean CVI across items. Generally, when the number of experts is six or more, and when the Item-SCV (I-SCV) is above 0.78 and the S-CVI is above 0.80, the content of the scale reflects well what is measured [41].

In this study, the S-CVI was 0.91 and the I-CVI ranged from 0.86 to 1.00, indicating good content validity of the scale.

#### (b) convergence validity

Convergence validity can be assessed in terms of the standardized factor loadings of each measured variable in the model relative to the latent variable, the average variance extracted (AVE) for each dimension, and the reliability. Table 2 shows that the standardized factor loadings of the measured variables ranged from 0.61 to 0.84, all above 0.50, indicating that each latent variable is highly representative of the topic to which it belongs [42]. In addition, the AVE of each latent variable was greater than 0.50 and the combined reliability CR was greater than 0.8, indicating good convergence validity [43].

#### (c) Discriminant validity

The dimensions were all significantly correlated with each other (*p* < 0.001) and the correlation coefficients between the dimensions were all less than the principal square root value of AVE except for F1 and F2 (Table 4). F1 and F2 are both subdimensions of ‘knowledge’ dimensions, so it is reasonable that the correlation coefficients between them are higher than the principal square root of AVE. When F1 and F2 are treated as one dimension to calculate the discriminant validity, the correlation coefficient is lower than the AVE’s principal square root. The above results suggest that the latent variables of this scale are not only somewhat correlated with each other but also differentiated from each other, indicating that the scale has good discriminant validity.

**Table 4:** The discriminant validity of each factor

	F1	F2	F3	F4		F1	F2	F3
**F1**	**1.00**	-	-	-	**F1**	**1.00**	-	-
**F2**	0.83 **	**1.00**	-	-	**F2**	0.36**	**1.00**	-
**F3**	0.38 **	0.33**	**1.00**	-	**F3**	0.57**	0.40**	**1.00**
**F4**	0.53 **	0.56**	0.39**	**1.00**	**the square root of AVE**	0.76	0.72	0.73
**the square root of AVE**	0.71	0.82	0.72	0.73	**When F1 and F2 are treated as one dimension**			

**p < 0.001, The lower triangle is the dimensional Pearson correlation; *AVE *The average variance extracted for each dimension

## Discussion

Snoring in childhood is often ignored by parents and may develop into childhood or adult OSAHS, which threatens their health seriously [44, 45]. Available assessment tools for children’s snoring can diagnose the disease through rapid screening [21, 22, 24]. Or it can facilitate the identification of snoring in children by raising knowledge of snoring in children among primary care physicians [26, 46], dentists [27], and pediatricians [47]. However, these tools are applied at the professional healthcare stage, neglecting the earlier presence of the family as the first line of defense in children’s healthcare. By raising the level of parental awareness of children’s snoring, parents will be able to change their attitudes and take a scientific approach to children’s snoring and promote the prevention or treatment of children’s snoring, thereby promoting children’s health. For example, when a child snores at night due to adenotonsillar hypertrophy [19], parents can realize the link between the two and bring the child for treatment on time. By making parents aware of the role that obesity plays in children’s snoring, parents will be able to focus on weight control in their children [48, 49]. This study developed and tested the reliability and validity of the first instrument to assess the level of knowledge, attitude, and practice of children’s parents in the community regarding Children Snoring. This scale is a valuable supplement to existing assessment tools for children snoring. It takes the parents of snoring children as the starting point and focuses on the value of the family in the prevention, diagnosis, and treatment of children snoring. This scale will help clinicians and researchers to determine the content and focus of their future educational work.

The scale consists of 25 items covering parental basic cognition toward Children Snoring, knowledge of complications, and attitudes and practices towards Children Snoring. Factor analyses support the subscale structure and we present preliminary evidence supporting the psychometric properties of the scale.

Since there is no validated scale for the parental perception of Children Snoring, the calibration validity test was not conducted in this study.

### Limitations

The limitations of this study are that firstly we recruited participants from Henan Province, China. Future research should evaluate the psychometric properties of this measure in other regions and countries. Finally, this study lacks other scales as external criteria to assess the criterion validity of this scale. Future research should explore criterion validity.

## Data Availability

The datasets generated during and/or analyzed during the current study are available from the corresponding author upon reasonable request.

## References

[1] Boss EF, Links AR, Saxton R, Cheng TL, Beach MC (2017). Parent experience of care and decision making for children who snore. JAMA Otolaryngol Neck Surg.

[2] Ali Khan I (2022). Role of adenotonsillectomy and tonsillectomy in children with down syndrome who develop obstructive sleep apnea by obesity as a risk factor. Menahem S, editor. Int J Pediatr.

[3] Xu Z, Wu Y, Tai J, Feng G, Ge W, Zheng L (2020). Risk factors of obstructive sleep apnea syndrome in children. J Otolaryngol Head Neck Surg.

[4] Zhao Z, Zheng L, Huang X, Li C, Liu J, Hu Y (2021). Effects of mouth breathing on facial skeletal development in children: a systematic review and meta-analysis. BMC Oral Health.

[5] Arens R, Marcus CL (2004). Pathophysiology of upper airway obstruction: a developmental perspective. Sleep.

[6] Katila M, Saarenpää-Heikkilä O, Saha MT, Vuorela N, Huhtala H, Korhonen LS (2021). Prevalence and evolution of snoring and the associated factors in two-year-old children. Sleep Med.

[7] Li AM, Zhu Y, Au CT, Lee DLY, Ho C, Wing YK (2013). Natural history of primary snoring in school-aged children. Chest.

[8] Brockmann PE, Bruni O, Kheirandish-Gozal L, Gozal D (2020). Reduced sleep spindle activity in children with primary snoring. Sleep Med.

[9] Collado MC, Katila MK, Vuorela NM, Saarenpää-Heikkilä O, Salminen S, Isolauri E (2019). Dysbiosis in snoring children: an interlink to comorbidities?. J Pediatr Gastroenterol Nutr.

[10] Esteller E, Villatoro JC, Agüero A, Lopez R, Matiñó E, Argemi J (2018). Obstructive sleep apnea syndrome and growth failure. Int J Pediatr Otorhinolaryngol.

[11] Horne RSC (2020). Endothelial damage in children with sleep-disordered breathing. Am J Respir Crit Care Med.

[12] Kim KM, Kim JH, Kim D, Lim MH, Joo H, Yoo SJ (2020). Associations among high risk for sleep-disordered breathing, related risk factors, and attention Deficit/Hyperactivity symptoms in elementary school children. Clin Psychopharmacol Neurosci.

[13] Kontos A, Willoughby S, Lushington K, Martin J, Wabnitz D, Dorrian J (2020). Increased platelet aggregation in children and adolescents with sleep-disordered breathing. Am J Respir Crit Care Med.

[14] Roux F, D’Ambrosio C, Mohsenin V (2000). Sleep-related breathing disorders and cardiovascular disease. Am J Med.

[15] Gunnlaugsson S, Abul MH, Wright L, Petty CR, Permaul P, Gold DR, et al. Associations of Snoring and Asthma Morbidity in the School Inner-City Asthma Study. J Allergy Clin Immunol Pract. 2021;9(10):3679–3685.e1. 10.1016/j.jaip.2021.05.022 PMC8511301 34102347

[16] Tan HL, Alonso Alvarez ML, Tsaoussoglou M, Weber S, Kaditis AG (2017). When and why to treat the child who snores? Treatment indications for pediatric OSAS. Pediatr Pulmonol.

[17] Yu PK, Radcliffe J, Gerry Taylor H, Amin RS, Baldassari CM, Boswick T (2022). Neurobehavioral morbidity of pediatric mild sleep-disordered breathing and obstructive sleep apnea. Sleep.

[18] Nieminen P, Tolonen U, Löppönen H (2000). Snoring and obstructive sleep apnea in children: a 6-month follow-up study. Arch Otolaryngol Neck Surg.

[19] Strocker AM, Shapiro NL (2007). Parental understanding and attitudes of pediatric obstructive sleep apnea and adenotonsillectomy. Int J Pediatr Otorhinolaryngol.

[20] Zhai F, Liu X, Yan F, Ma Q (2016). Investigation of the cognitive status and the need of health education on children snoring from the parents whose children were in hospital by snoring. Chin J Pract Nurs..

[21] Soh HJ, Rowe K, Davey MJ, Horne RSC, Nixon GM (2018). The OSA-5: development and validation of a brief questionnaire screening tool for obstructive sleep apnea in children. Int J Pediatr Otorhinolaryngol.

[22] Bruni O, Ottaviano S, Guidetti V, Romoli M, Innocenzi M, Cortesi F (1996). The Sleep Disturbance Scale for Children (SDSC) construct ion and validation of an instrument to evaluate sleep disturbances in childhood and adolescence. J Sleep Res.

[23] Meltzer LJ, Biggs S, Reynolds A, Avis KT, Crabtree VM, Bevans KB (2012). The children’s report of sleep patterns – sleepiness scale: a self-report measure for school-aged children. Sleep Med.

[24] Chervin RD, Hedger K, Dillon JE, Pituch KJ (2000). Pediatric sleep questionnaire (PSQ): validity and reliability of scales for sleep-disordered breathing, snoring, sleepiness, and behavioral problems. Sleep Med.

[25] Uong EC, Jeffe DB, Gozal D, Arens R, Holbrook CR, Palmer J (2005). Development of a measure of knowledge and attitudes about obstructive sleep apnea in children (OSAKA-KIDS). Arch Pediatr Adolesc Med.

[26] Devaraj NK (2020). Knowledge, attitude, and practice regarding obstructive sleep apnea among primary care physicians. Sleep Breath.

[27] Bian H, Smith CL (2006). Development of a questionnaire to assess dentists’ knowledge, opinion, education resources, physician cooperation, and clinical practice regarding obstructive sleep apnea (OSAQ-D). Sleep Breath.

[28] Bettinghaus EP (1986). Health promotion and the knowledge-attitude-behavior continuum. Prev Med.

[29] Liu J, Qiu H, Zhang X, Zhang C, He F, Yan P (2023). Development of billing post competency evaluation index system for nurses in China: a Delphi study. BMC Nurs.

[30] Zobdeh A, Bandari R, Heravi-Karimooi M, Mashayekh M, Hazrati M, Montazeri A (2023). Development and validation of the short form domestic elder abuse assessment questionnaire (SF-DEAQ). BMC Geriatr.

[31] Shrestha N (2020). Detecting multicollinearity in regression analysis. Am J Appl Math Stat.

[32] Haitovsky Y (1969). Multicollinearity in regression analysis: comment. Rev Econ Stat.

[33] Gulliksen H (1945). The relation of item difficulty and inter-item correlation to test variance and reliability. Psychometrika.

[34] Piedmont RL, Hyland ME (1993). Inter-item correlation frequency distribution analysis: a method for evaluating scale dimensionality. Educ Psychol Meas.

[35] Diamantopoulos A, Sarstedt M, Fuchs C, Wilczynski P, Kaiser S (2012). Guidelines for choosing between multi-item and single-item scales for construct measurement: a predictive validity perspective. J Acad Mark Sci.

[36] Demirtaş A, Akbayrak N (2017). Development of an assessment scale for treatment compliance in type 2 diabetes Mellitus in Turkish population: psychometric evaluation. Int J Nurs Sci.

[37] Chen X, Luo L, Jiang L, Shi L, Yang L, Zeng Y (2021). Development of the nurse’s communication ability with angry patients scale and evaluation of its psychometric properties. J Adv Nurs.

[38] Juhola J, Arokoski JPA, Ervasti J, Kivimäki M, Vahtera J, Myllyntausta S (2021). Internal consistency and factor structure of Jenkins Sleep Scale: cross-sectional cohort study among 80 000 adults. BMJ Open.

[39] Wu BJ, Lan TH, Hu TM, Lee SM, Liou JY (2015). Validation of a five-factor model of a Chinese Mandarin version of the positive and negative syndrome scale (CMV-PANSS) in a sample of 813 schizophrenia patients. Schizophr Res.

[40] Shrestha N (2021). Factor analysis as a tool for survey analysis. Am J Appl Math Stat.

[41] Polit DF, Beck CT, Owen SV (2007). Is the CVI an acceptable indicator of content validity? Appraisal and recommendations. Res Nurs Health.

[42] Els R, Meyer H, Ellis S (2022). A measurement scale developed to investigate the effect of leaders’ perceptions regarding attitudes towards and commitment to quality management of training. Int J Train Dev.

[43] Sarstedt M, Ringle CM, Hair JF, Homburg C, Klarmann M, Vomberg A (2022). Partial least squares structural equation modeling. Handbook of market research.

[44] Zeng G, Xu G, Liu H, yu, Gao Z (2022). Association between mean platelet volume and obstructive sleep apnea-hypopnea syndrome in children. Medicine (Baltimore).

[45] Swami H, Anand V (2017). Effect of cephalometric variables in paediatric snorers. Int J Otorhinolaryngol Head Neck Surg.

[46] Erichsen D, Godoy C, Gränse F, Axelsson J, Rubin D, Gozal D (2012). Screening for sleep disorders in pediatric primary care: are we there yet?. Clin Pediatr (Phila).

[47] Tamay Z, Akcay A, Kilic G, Suleyman A, Ones U, Guler N (2006). Are physicians aware of obstructive sleep apnea in children?. Sleep Med.

[48] Spruyt K, Capdevila OS, Serpero LD, Kheirandish-Gozal L, Gozal D (2010). Dietary and physical activity patterns in children with obstructive sleep apnea. J Pediatr.

[49] Sekine M, Yamagami T, Hamanishi S, Handa K, Saito T, Nanri S (2002). Parental obesity, lifestyle factors and obesity in preschool children: results of the Toyama Birth Cohort Study. J Epidemiol.

